# Identification of a novel angiogenic peptide from periostin

**DOI:** 10.1371/journal.pone.0187464

**Published:** 2017-11-02

**Authors:** Ba Reun Kim, Yang Woo Kwon, Gyu Tae Park, Eun Jung Choi, Jeong Kon Seo, Il Ho Jang, Seung-Chul Kim, Hyun-Chang Ko, Sang Chul Lee, Jae Ho Kim

**Affiliations:** 1 Department of Physiology, School of Medicine, Pusan National University, Yangsan, Republic of Korea; 2 UNIST Central Research Facility, Ulsan National Institute of Science and Technology, Ulsan, Republic of Korea; 3 Department of Oral Biochemistry and Molecular Biology, School of Dentistry, Pusan National University, Yangsan, Republic of Korea; 4 Department of Obstetrics and Gynecology, School of Medicine, Pusan National University, Yangsan, Republic of Korea; 5 Department of Dermatology, School of Medicine, Pusan National University, Yangsan, Republic of Korea; 6 Functional Genomics Research Center, KRIBB, Daejeon, Republic of Korea; 7 Research Institute of Convergence Biomedical Science and Technology, Pusan National University Yangsan Hospital, Yangsan, Republic of Korea; Medical University Innsbruck, AUSTRIA

## Abstract

Angiogenic peptides have therapeutic potential for the treatment of chronic ischemic diseases. Periostin, an extracellular matrix protein expressed in injured tissues, promotes angiogenesis and tissue repair. We previously reported that *in vivo* administration of both recombinant full-length protein and the first FAS I domain of periostin alleviated peripheral artery occlusive disease by stimulating the migration of humane endothelial colony forming cells (ECFCs) and subsequent angiogenesis. In the present study, we ascertained the peptide sequence responsible for the periostin-induced angiogenesis. By serial deletion mapping of the first FAS I domain, we identified a peptide sequence (amino acids 142–151) of periostin for stimulation of chemotactic migration, adhesion, proliferation and endothelial tube formation of human ECFCs *in vitro*. Chemotactic migration of ECFCs induced by the periostin peptide was blocked by pre-incubation with an anti-β5 integrin neutralizing antibody. Treatment of ECFCs with the periostin peptide led to phosphorylation of both AKT and ERK, and pretreatment of ECFCs with the MEK-ERK pathway inhibitor U0126 or the PI3K-AKT pathway inhibitors, LY294002 or Wortmannin, blocked the periostin peptide-stimulated migration of ECFCs. These results suggest that the synthetic periostin peptide can be applied for stimulating angiogenic and therapeutic potentials of ECFCs.

## Introduction

Angiogenesis is defined as the formation of new capillaries from pre-existing blood vessels[1],. Formation of a new vasculature is essential for the removal of debris, providing nutrients and oxygen to injured tissues. Therefore, angiogenesis is a critical step for repair of injured tissues, and insufficient angiogenesis can result in impaired wound healing and chronic wound formation [2]. Accumulating evidence suggests that chronic wound recovery is promoted by application of topical cytokines and growth factors [3, 4]. However, expression and purification of recombinant cytokines for therapeutic drug development require high manufacturing costs and raise safety issues. To develop recombinant proteins for clinical application, improved stability and cost efficacy are required. Instead, development of synthetic small peptides to stimulate wound healing processes will be useful for treatment of chronic wounds. Therefore, it is necessary to identify new angiogenic peptides for clinical applications.

There are more than 7000 naturally occurring peptides, and these are pivotal in human physiology, including actions as hormones, neurotransmitters, growth factors, ion channel ligands, or anti-infection [5–8]. Given their attractive pharmacological profile and intrinsic properties, peptides represent an excellent starting point for the design of novel therapeutics. Their specificity translates into excellent safety, tolerability, and efficacy profiles in humans. Furthermore, peptide therapeutics is associated with lower production complexity compared with protein-based biopharmaceuticals, and therefore, the production costs are lower, generally approaching those of small molecules. As such, peptides are in the sweet spot between small molecules and biopharmaceuticals [9]. Peptides are naturally degraded in the blood stream by circulating enzymes to their component amino acids. As these are natural biological products, peptide drugs are associated with less accumulation in body tissue and less toxicity owing to the low doses.

Periostin, originally named osteoblast-specific factor 2 (OSF2), was first identified in a mouse osteoblastic cell line as a cell adhesion protein and was recently classified as a novel matricellular protein, sharing a homology with the insect cell adhesion molecule fasciclin 1 (FAS-1) [10, 11]. Periostin is involved in the regulation of cell adhesion and motility through an integrin-dependent mechanism [12]. Periostin functions are associated with skeletal development, heart development, and cancer [13–15]. Furthermore, periostin promotes angiogenesis, migration, and invasion of human umbilical endothelial cells [16] and stimulates tumor angiogenesis in breast cancer [17]. In a previous study, we reported that recombinant periostin protein stimulated the migration and tube formation of ECFCs. After mapping the functional domains of periostin associated with angiogenesis, the first FAS-I domain of periostin has been identified to possess angiogenic potential as potent as the whole periostin [18]. As the first FAS I domain of periostin is a recombinant protein, there are limits for its therapeutic application. Therefore, it is essential to identify peptide sequence responsible for angiogenic activity of periostin.

In the present study, we identified an angiogenic peptide by serial mapping of the first FAS I domain of periostin. Our results showed that the periostin-derived peptide stimulated the migration, tube formation, and proliferation of ECFCs. Our finding may contribute to the development of a novel peptide drug to promote angiogenesis by regulating ECFC activity.

## Materials and methods

### Materials

Fetal bovine serum and trypsin were purchased from Invitrogen (Carlsbad, CA). Endothelial Growth Medium-2 bullet kit was purchased from Lonza (Basel, Swiss). Culture plates were purchased from Nunc (Roskilde, Denmark). A recombinant human periostin protein was purchased from R&D Systems, Inc. (Minneapolis, MN). Anti-CD31 rat antibody (MEC 13.3) and growth factor-reduced Matrigel™ were purchased from BD Biosciences (Franklin Lakes, NJ). Rabbit anti-α-SMA antibody was purchased from Abcam PLC (Cambridge, UK). QuikChange II Site-Directed Mutagenesis Kit was purchased from Agilent Technologies (Santa Clara, CA). Glyceraldehyde-3-Phosphate Dehydrogenase antibody (MAB374) and U0126 were purchased from Millipore (Temecula, CA). Antibodies against AKT (#9272S), p-AKT (#3787S), ERK (#9102), and p-ERK (#9101) were purchased from Cell Signaling Technology (Boston, MA). LY294002, Wortmannin, Trypsin/EDTA, Ponceau S solution, and monoclonal anti-α-SMA antibody (A2547) were purchased from Sigma-Aldrich (Saint Louis, MO). Peroxidase-labeled secondary antibodies and Enhanced Chemiluminescence Western blotting system were purchased from Amersham Biosciences (Piscataway, NJ). The synthetic peptides were synthesized at Anygen (Kwangju, Republic of Korea, www.anygen.com) and Chinapeptides (Shanghai, China, http://www.chinapeptides.com). The purity of synthesized peptides was >98%.

### Serial deletion mapping of recombinant periostin protein

For the expression of functional domains of the first FAS-I domain of periostin as recombinant His-tagged proteins, serial deletion mutants of the first FAS-I domain were sub-cloned into a pET-30a expression vector (Novagen, Madison, WI) by ligation of PCR-generated cDNA fragments containing additional EcoRI and Hind III sites into the pET-30a vector. The amino acid sequences of the six fragments derived from the first FAS-I domain of periostin are as follows: amino acid 97~216, 97~196, 97~176, 97~156, 97~136, and 97~116. Site-directed mutagenesis of the first FAS-I domain of periostin cDNA was performed using a QuikChange kit (Stratagene). The primer sequences for site directed mutagenesis are as follows: 97~216 (5'-GTGGGAGCCACCACAACGCAGAAGCTTGCGGCCGCACTCGAG-3'), 97~196 (5'- GAGTAATGAGGCTTGGGACAACAAGCTTGCGGCCGCACTCGAG-3'), 97~176 (5'- CTGATATCCGTAGAGGTTTGGAGAAGCTTGCGGCCGCACTCGAG-3'), 97~156 (5'-CACATGATTAATAAGAGAATGAAGCTTGCGGCCGCACTCGAGCAC-3'), 97~136 (5'- CAATGTATAACAATTTGGGGCTTAAGCTTGCGGCCGCACTCGAG-3'), 97~116 (5'-GTTAATTGTGCTCGAATCATCCATGGGAAGCTTGCGGCCGCACTCGAG-3'). The mutations were verified by automatic DNA sequencing. His-tagged recombinant proteins were expressed in BL21 (DE3) cells, harvested, and purified using a nickel-NTA agarose column (Qiagen, Inc., Valencia, CA) as described in the instruction manual. Endotoxin was removed with Detoxi-Gel Endotoxin Removing Gel (Pierce, Rockford, IL), and the removal of endotoxin was confirmed by Limulus Amebocyte Lysate test (Cape Cod, East Falmouth, MA). The purified recombinant proteins were loaded onto 15% sodium dodecyl sulfate-acrylamide gel for electrophoresis and silver staining.

### Cell culture

Human ECFCs were isolated from human umbilical cord blood and collected in disposable sterile pyrogen-free bags (Green Cross, Yongin, Korea) containing anticoagulant. Written informed consent was obtained from all donors and the study was approved by the Institutional Review Board of Pusan National University Hospital. Mononuclear cells were isolated from the blood with Histopaque-1077 (Sigma-Aldrich, Switzerland) as described previously [19]. ECFCs were seeded on culture dishes coated with 0.1% gelatin (Sigma-Aldrich) and maintained in endothelial cell basal medium-2 (EBM-2) (Clonetics, San Diego, CA) supplemented with EGM-2 MV Single Quotes containing 5% fetal bovine serum (FBS), human VEGF-1, human fibroblast growth factor-2, human epidermal growth factor, insulin-like growth factor-1, and ascorbic acid. After four days in culture, non-adherent cells were removed and adherent cells were trypsinized and re-plated at a density of 1 × 10^6^ per well until day 7 [19].

### Cell migration assay

ECFC migration was assayed using a disposable 96-well chemotaxis chamber (Neuro Probe, Inc., Gaithersburg, MD). ECFCs were harvested with 0.05% trypsin containing 0.02% EDTA, washed once, and suspended in EBM-2 at a concentration of 1 × 10^5^ cells/ml. A membrane filter with 8-μm pores for the chemotaxis chamber was pre-coated overnight with 20 μg/ml rat-tail collagen at 4°C; an aliquot (50 μl) of ECFC suspension was loaded into the upper chamber, and EBM-2 supplemented with recombinant proteins of periostin was then placed in the lower chamber. Following incubation for 12 h at 37°C, the filters were disassembled, and the upper surface of each filter was scraped free of cells by wiping it with a cotton swab. The number of cells that migrated to the lower surface of each filter was determined by counting the cells in four locations under microscopy at × 100 magnification after staining with Hoechst.

To clarify the involvement of integrins in the periostin-stimulated migration of ECFCs, cells were pre-incubated with function-blocking monoclonal antibodies specific to integrins β1 (HMβ1–1, BioLegend), β3 (F11, BD), and β5 (P1F6, Millipore), or control antibody (each 5 μg/ml) at 37°C for 30 min. The antibody-incubated cells were then loaded into the upper chamber of a 96-well chemotaxis chamber pre-coated with rat-tail collagen, and migration of ECFCs was determined after incubation with recombinant periostin proteins for 12 h.

### Cell adhesion assay

Ninety-six-well microculture plates (Falcon, Becton-Dickinson, Mountain View, CA) were incubated with recombinant periostin proteins or recombinant periostin domains 1 to 5 at 37°C for 1 h, followed by blocking with PBS containing 0.2% BSA for 1 h at 37°C. Cells were trypsinized and suspended in the culture media at a density of 2 × 10^5^ cells/ml, and 0.1 ml of the cell suspension was then added to each well of the plate. Analysis of cell attachment was performed as follows. After incubation for 1 h at 37°C, unattached cells were removed by rinsing twice with PBS. The number of attached cells was determined by counting the cells under microscopy at × 100 magnification after staining with hematoxylin and eosin.

### Tube formation assay

For tube formation assay of ECFCs, aliquots (50 μl) of growth factor-reduced Matrigel (10 mg protein/ml) were added to 96-well culture dishes and polymerized for 30 min at 37°C. ECFCs were trypsinized, resuspended in EBM-2 basal medium supplanted with 1% FBS, and plated onto a layer of Matrigel at a density of 1 × 10^5^ cells/well. The cells were then exposed to EBM-2 media with 1% FBS or recombinant periostin domains 1 and periostin peptide. After incubation of the Matrigel cultures at 37°C overnight cells were labeled with 2 μM Calcein AM for 30 minutes at 37°C in 5% CO_2_ incubator. The cultures were photographed using 484 nm excitation and 520 nm emission filter on a fluorescent microscope equipped with × 10 objective [20]. The images of the tubes were scanned into Adobe Photoshop (version 7.0.1) and quantified using ImageJ software (National Institutes of Health)

### Cell proliferation assay

To assess the effects of peptides on cell proliferation, cells were seeded at 5×10^3^ cells per well (96-well plates, SPL) in 100 μl endothelial cell basal medium-2 supplemented with 0.5% FBS. Cells were treated with 0 to 0.5 μM peptide for 48 h. Relative cell numbers were determined by MTT (3-(4, 5-dimethylthiazol-2-yl) 2, 5-diphenyl tetrazolium bromide) assay. After 0, 24, 48 h of treatment, cells were stained with 100 μl sterile MTT dye (0.5 mg/ml, Sigma) for 2 h at 37°C, followed by removal of the culture medium and addition of 100 μl DMSO. Absorbance was measured at 570 nm, with 655 nm as the reference wavelength using a micro-plate spectrophotometer (Tecan, Morrisville, NC). The relative percentage of cell viability was calculated by dividing the absorbance of treated cells by that of the control in each experiment.

### Western blotting

Cells were lysed in lysis buffer (20 mM Tris-HCL, 1 mM EGTA, 1 mM EDTA, 10 mM NaCl, 0.1 mM phenylmethylsulfonyl fluoride, 1 mM Na_3_VO_4_, 30 mM sodium pyrophosphate, 25 mM β-glycerol phosphate, 1% Triton X-100, pH 7.4). Cell lysates were centrifuged at 15,000 rpm for 15 min at 4°C, and supernatants were used for Western blotting. Lysates were resolved by sodium dodecyl sulfate-polyacrylamide gel electrophoresis (SDS-PAGE), transferred onto a nitrocellulose membrane, and stained with 0.1% Ponceau S solution (Sigma- Aldrich). After blocking with 5% non-fat milk, the membranes were immunoblotted with various antibodies overnight, and the bound antibodies were visualized with horseradish peroxidase-conjugated secondary antibodies, using the Enhanced Chemiluminescence Western blotting system (ECL, Amersham Biosciences).

### Statistical analysis

Results of multiple observations are presented as the mean ± SD. For multivariate data analysis, group differences were assessed using one-way or two-way analysis of variance, followed by post hoc comparisons tested using Scheffe’s method.

## Results

### Identification of a pro-angiogenic peptide sequence of periostin

**Fig 1:**
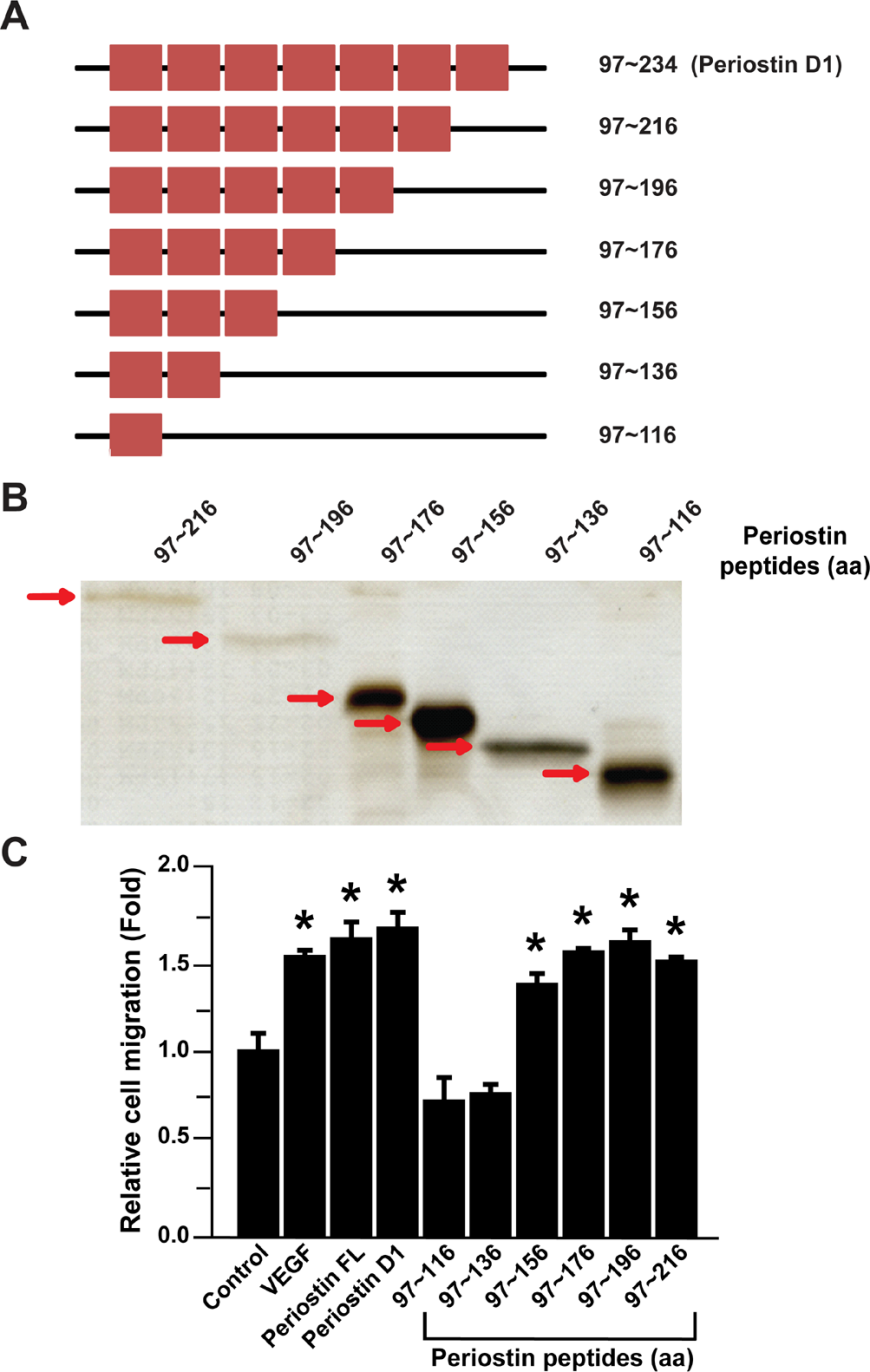
Effects of the periostin domain 1 and its fragments on migration of ECFCs. (A) Schematic diagram of human periostin D1 domain (amino acid 97~234) and its six truncated protein constructs (amino acids 97~216, 97~196, 97~176, 97~156, 97~136, 97~116). The red square box indicates 20 amino acid of the periostin amino acid sequence. (B) Purification and SDS-PAGE analysis of the recombinant fragments of periostin D1 domain. (C) Effects of the Periostin D1 domain and its fragments on migration of ECFCs. Chemotactic migration of ECFCs was determined after treatment with VEGF, full length recombinant periostin, the periostin D1 domain or its fragments (each 10 μg/ml). Data represent mean ± S.D. (n = 9), * indicates *P* < 0.05 vs. control.

To identify the functional peptide sequence involved in periostin-stimulated angiogenesis, the first FAS-I domain of periostin (amino acid 97~234) was divided into seven fragments containing 20 amino acids. The recombinant proteins were over-expressed as His-tagged proteins in Escherichia coli and purified using Ni-NTA affinity chromatography. The purity of the purified seven fragments was estimated to be approximately 90% (Fig 1A and 1B). We next performed a Transwell migration assay to examine the effects of the periostin-derived peptide fragments on angiogenic activity of ECFCs. As shown Fig 1C, migration ability of ECFCs was not affected by treatment with the periostin peptide covering amino acid 97~116 or 97~136. Migration of ECFCs was significantly activated by periostin peptides covering amino acid 97~156, 97~176, 97~196, and 97~216. These results suggest that periostin peptide covering 136~156 amino acid is responsible for the periostin-induced migration of ECFCs (S1 Fig).

### Enhanced migration and tube formation of ECFCs by the periostin peptide 136~151

**Fig 2:**
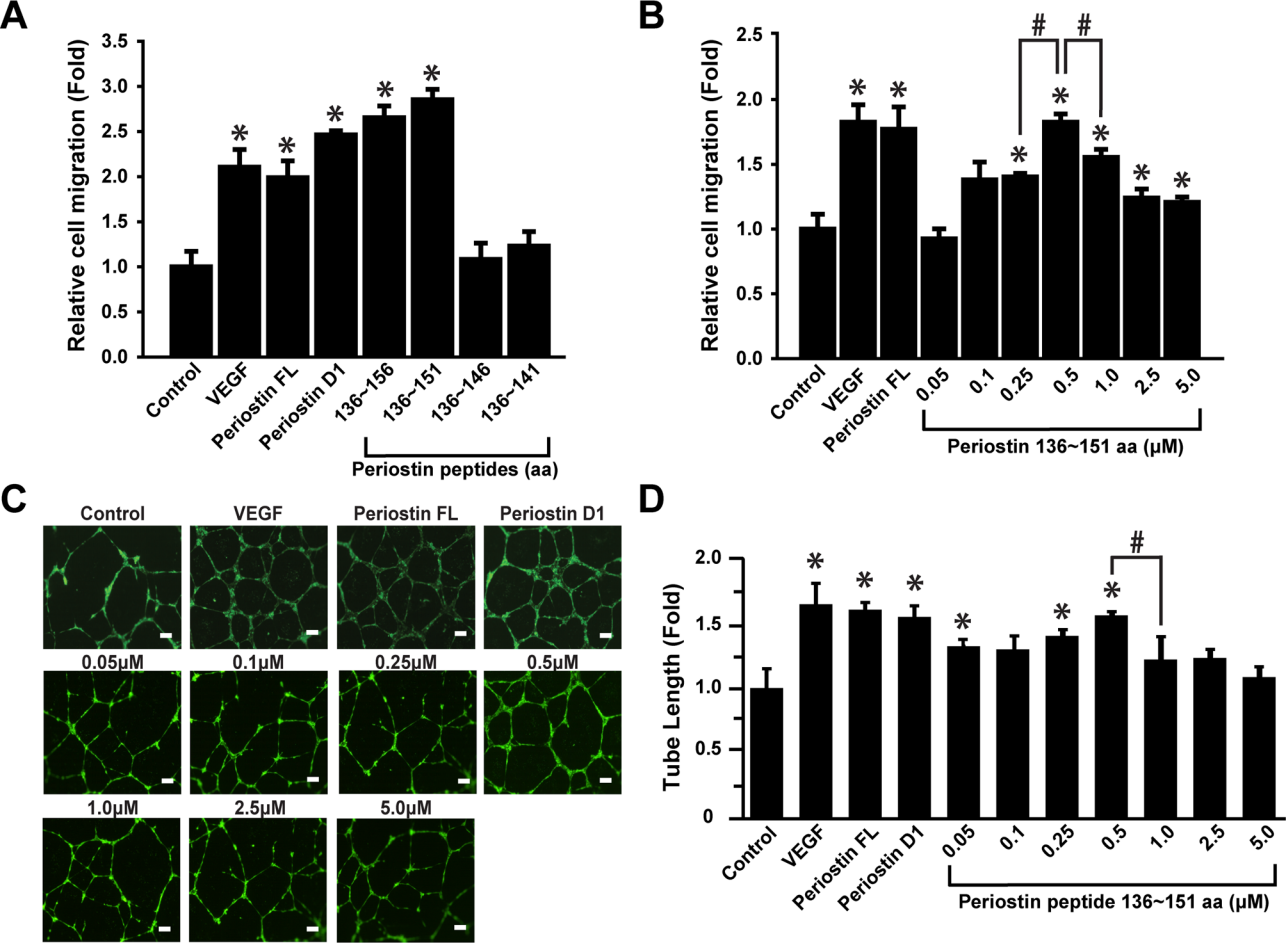
Effects of the periostin peptide 136~156 and its fragments on migration and tube formation of ECFCs. (A) Effects of the periostin peptide 136~151 and its fragments on chemotactic migration of ECFCs. (B) Dose-dependent effects of the periostin peptide 136 ~ 151 on chemotactic migrations of ECFCs. Migration of ECFCs in response to increasing concentrations of the periostin peptide 136~151 was determined. (C) Effects of the periostin peptide 136~151 on the tube formation of ECFCs (scale bar = 100 μm). (D) Tube formation was quantified by measuring the length of tubes in four random fields from each well and normalizing the values relatives to those of the corresponding control. Data represent mean ± S.D. (n = 12), #, P <0.05; * indicates *P* < 0.05 vs. control.

To confirm the result that periostin peptide 136~156 stimulates migration of ECFCs, we synthesized the periostin peptide 136~156 and the effect of periostin peptide on cell migration was explored. As shown in Fig 2A, periostin peptide 136~156 stimulated migration of ECFCs. To further characterize the minimal sequence of periostin peptide, we synthesized several peptides covering amino acid 136~151, 136~146, and 136~141. Migration of ECFCs was stimulated by periostin peptide 136~151, but not by 136~146 or 136~141. The periostin peptide 136~151 dose-dependently stimulated migration of ECFCs with a maximal stimulation at 0.5 μM (Fig 2B). In addition, endothelial tube formation of ECFCs was significantly induced in response 0.5 μM periostin peptide 136~151 treatment, as potent as VEGF or periostin D1 domain (Fig 2C and 2D). Altogether, these results suggest that periostin peptide 136~151 is responsible for migration and tube formation of ECFCs.

### Identification of a minimal pro-angiogenic peptide sequence by serial deletion mapping of periostin peptide 136~151

**Fig 3:**
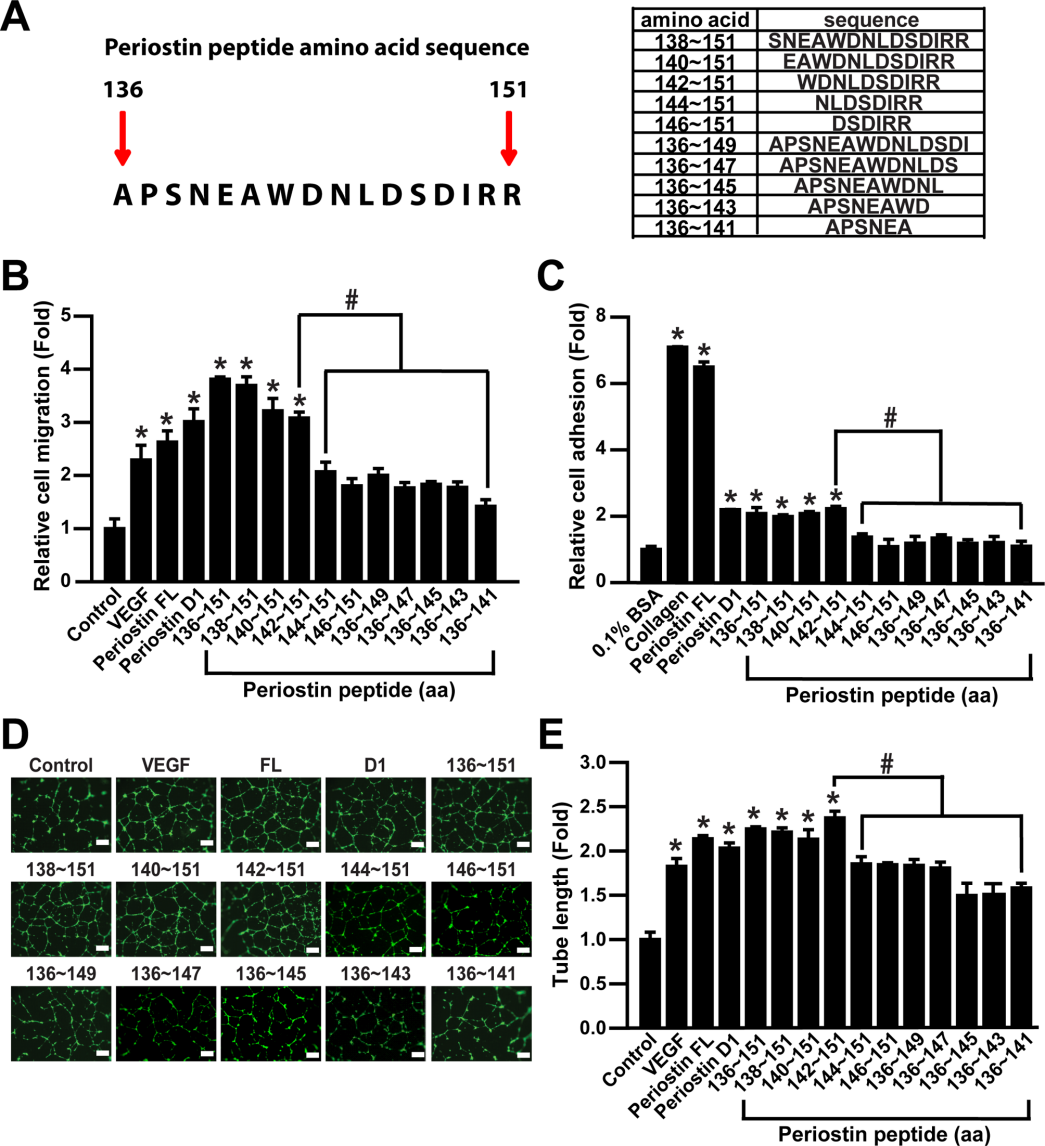
Effects of the periostin peptide 136~151 and its fragments on angiogenic activities of ECFCs *in vitro*. (A) Amino acid sequence of the periostin peptide 136~151. (B, C) Effects of periostin peptide 136~151 and its fragments on migration (B) and adhesion (C) of ECFCs. Migration and adhesion of ECFCs were measured in response to VEGF, recombinant periostin FL (10 μg/ml), the periostin D1 domain (10 μg/ml), or various fragments of periostin 136~151 (each 0.5 μM) after treatment for 12 h. (D, E) Effects of various periostin peptides on tube formation of ECFCs. The length of tubes was quantified in four random fields from each well and the values was normalized relative to those of the corresponding control (scale bar = 100 μm). Data are expressed as mean ± SD. #, P <0.05; * indicates *P* < 0.05 vs. control.

To discover the minimum pro-angiogenic sequence of periostin peptide, we synthesized eleven peptides deleted from N-terminal or C-terminal ends of periostin peptide 136~151 (Fig 3A). Deletion of C-terminal end of periostin peptide 136~151 markedly inhibited ECFC migration, suggesting an essential role of C-terminal end in periostin peptide 136~151 (Fig 3B). Moreover, N-terminal deletion of the periostin peptide 136~151 from amino acid 144 attenuated ECFC migration. Treatment of ECFCs with the periostin peptide 142~151 but not 144~151 stimulated ECFC migration, suggesting a pivotal role of peptide 142~151 on the periostin D1 domain-induced ECFC migration. In a previous study, coating with the periostin D1 domain, but not other periostin domains, resulted in significant enhancement of the adhesive capacity of ECFCs [18]. Consistent with the periostin peptide-induced migration, not only the periostin D1 domain but also the periostin peptide 142~151 stimulated adhesion of ECFCs (Fig 3C). To confirm the angiogenic activities of the periostin peptides, we measured the effects of periostin peptides on the tube-forming capacity of ECFCs in Matrigel-coated dishes. Both N-terminal deletion of periostin peptide 136~151 from amino acid 144 and C-terminal deletion of the periostin peptide 136~151 markedly attenuated the tube forming activity of ECFCs (Fig 3D and 3E). These results strongly suggest that the periostin peptide 142~151 exhibits angiogenic activities stimulating migration, adhesion, and tube formation of human ECFCs.

### Periostin peptide 142~151-stimulated proliferation of ECFCs

**Fig 4:**
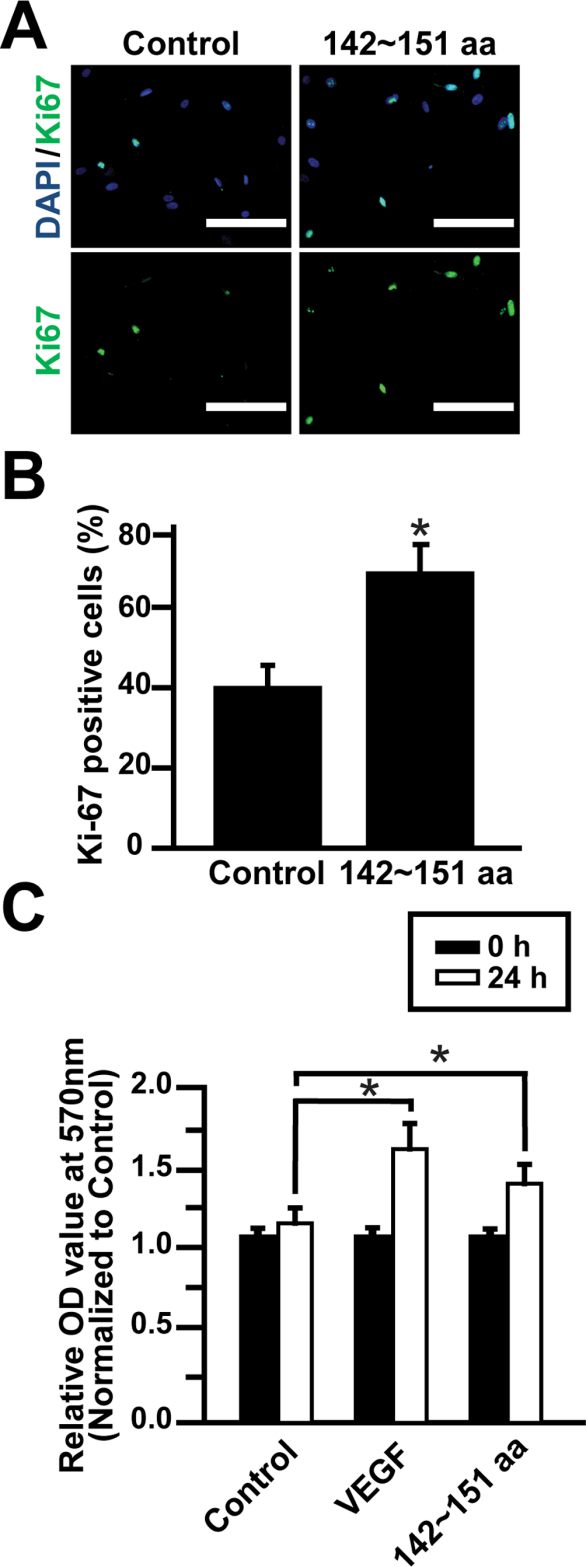
Effect of periostin peptide 142~151 on ECFC proliferation. **(A, B)** ECFCs were incubated with the periostin peptide 142~151 for 24 h and labeled with anti-Ki67 antibody (green) and nuclei were counterstained with DAPI (blue). The number of Ki67-positive cells was quantified per DAPI. (scale bar = 50 μm). (C) Effects of the periostin peptide 142~151 on proliferation of ECFCs. Human ECFC were treated with the periostin peptide 142~151 and VEGF for 24 h, followed by MTT analysis. Data are expressed as mean ± SD. * indicates *P* < 0.05.

To investigate whether the periostin peptide 142~151 can affect proliferative ability of ECFCs, expression of Ki-67, a marker for proliferating cells, was probed by immunocytochemistry after treatment with the periostin peptide 142~151 for 24 h. As shown in Fig 4A, treatment of ECFCs with the periostin peptide 142~151 significantly increased the number of Ki-67-positive cells. Treatment with the periostin peptide 142~151 increased the percentage of Ki67-positive cells from approximately 40% to over 70% (Fig 4B). We next investigated the possibility that the periostin peptide 142~151 might confer a selective advantage to human ECFCs by affecting their proliferation rate. We tested this possibility *in vitro* by conducting MTT assays. As shown in Fig 4C, the periostin peptide 142~151 stimulated proliferation of human ECFCs as potent as VEGF. These findings suggest that periostin peptide 142~151 could effectively contribute to the proliferation of ECFCs.

### Role of integrin β5 in the periostin peptide 142~151-induced migration of ECFCs

**Fig 5:**
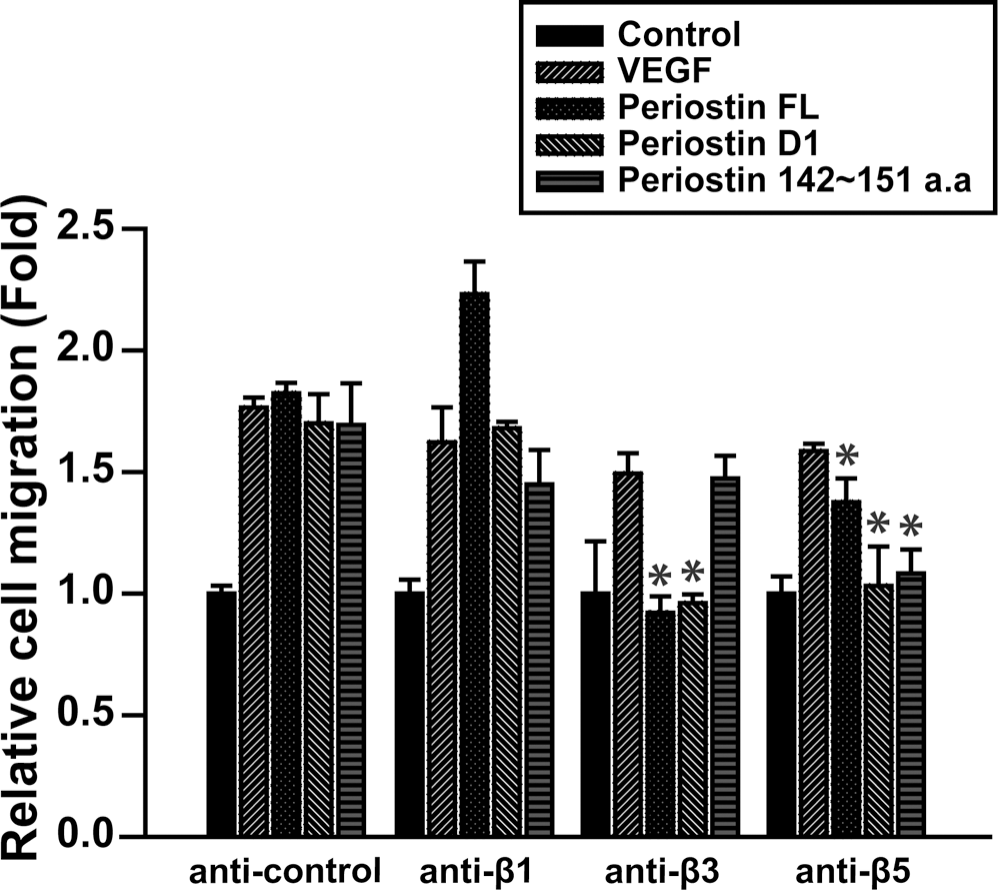
Effects of neutralizing antibodies against integrins β1, β3, and β5 on ECFC migration. ECFCs were pre-incubated with control antibody or neutralizing antibodies against integrins β1, β3, and β5, followed treatment with periostin FL (10 μg/ml), Periostin D1 (10 μg/ml), or 0.5 μM the periostin peptide 142~151 for determination of cell migration. Data represent mean ± S.D. (n = 6). * indicate *P* <0.05 vs. anti-control.

Periostin and the first FAS-I domain of periostin stimulate angiogenic activities of ECFCs by activation of integrins β3 and β5 [18]. To explore the involvement of integrins in periostin peptide 142~151-stimulated cellular responses, ECFCs were pre-incubated with neutralizing antibodies against β1, β3 and β5 integrins, followed by measurement of cell migration in response to the VEGF, full length periostin, the first FAS-I domain of periostin, and the periostin peptide 142~151. Pre-incubation of human ECFCs with an anti-integrin β5 antibody resulted in markedly attenuated cell migration stimulated by the periostin peptide 142~151; however, incubation with anti-β1 and -β3 antibodies did not block the periostin peptide 142~151-stimulated human ECFC migration (Fig 5). This suggests involvement of β5 integrin in human ECFC migration induced by the periostin peptide 142~151.

### Role of PI3K-AKT and MEK-ERK signaling pathways in the periostin peptide 142~151-stimulated migration of ECFCs

**Fig 6:**
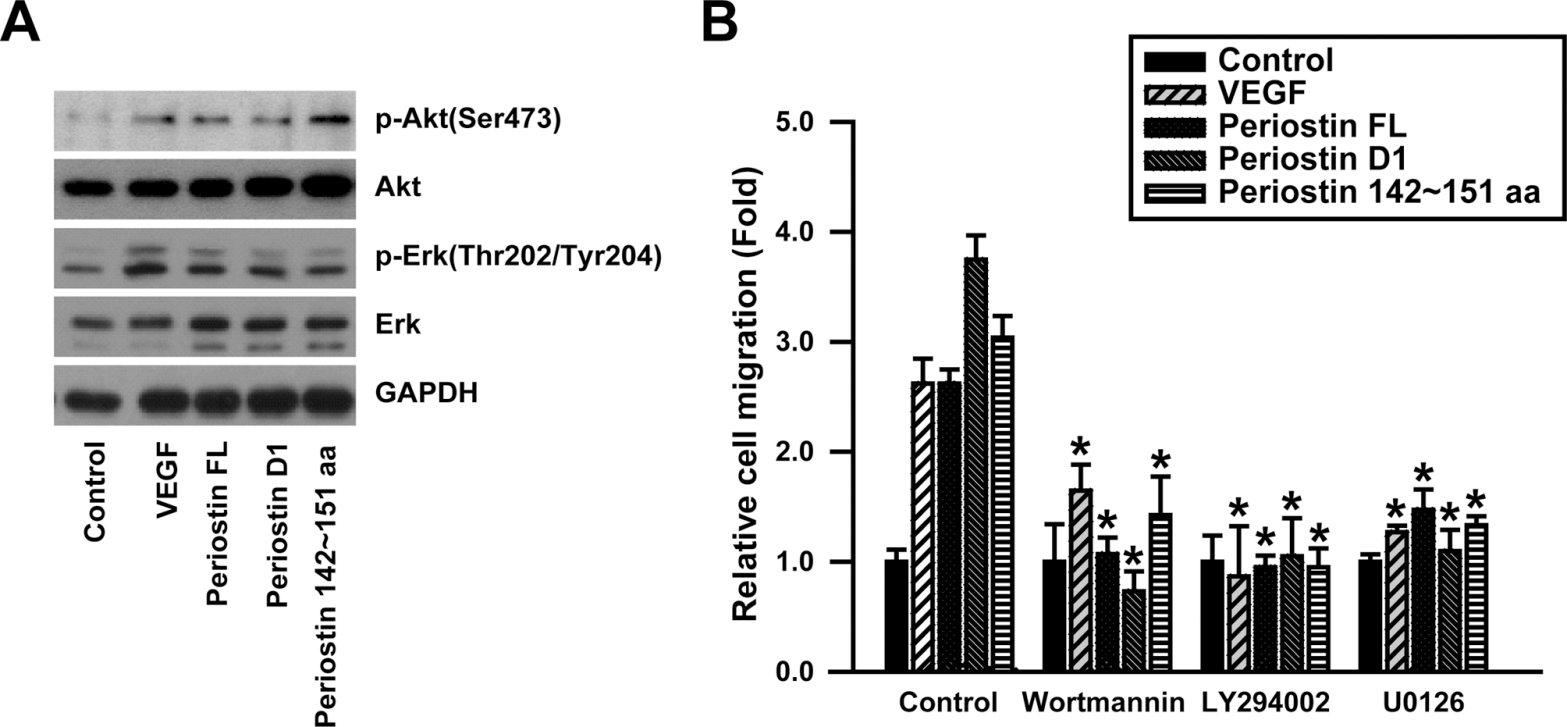
Role of PI3K and MEK1/2 pathway in the periostin peptide 142~151-induced migration of ECFCs. (A) Effects of the periostin peptide 142~151 on phosphorylation (Akt^Ser473^ and Erk^Thr202/Tyr204^) and expression levels of Akt and Erk were determined by Western blot analysis. The cells were treated with VEGF (10 ng/ml), the periostin FL (10 μg/ml), periostin D1 domain 1 (10 μg/ml), or the periostin 142~151 amino acid peptide (0.5 μM) for 1 minutes. Representative data from three independent experiments. (B) ECFCs were pre-incubated with the PI3K inhibitors Wortmannin and LY294002 or the ERK inhibitor U0126, followed by treatment with VEGF (10 ng/ml), periostin FL (10 μg/ml), the periostin D1 domain (10 μg/ml), or the periostin peptide 142~151 (0.5 μM). Data represent mean ± S.D. (n = 6). * indicate *P* <0.05 vs. control.

Periostin induces activation of Akt, Erk, and focal adhesion kinase-mediated signaling pathways. To clarify the role of Akt and Erk in the periostin peptide 142~151-induced cellular responses, the phosphorylation levels of Akt and Erk were examined in human ECFCs. As shown in Fig 6A, treatment with the periostin peptide 142~151 increased the phosphorylation levels of Akt and Erk as potent as full length and the first FAS-I domain of periostin. We next examined the effects of chemical inhibitors specific for PI3K-Akt and MEK-ERK pathways on migration of ECFCs. Pre-treatment of human ECFCs with LY294002 and Wortmannin, the PI3K-specific inhibitors, MEK-specific inhibitor U0126 blocked the cell migration stimulated by the periostin peptide 142~151 (Fig 6B). These results suggest that PI3K-Akt and MEK-ERK pathways play a pivotal role in the human ECFC migration stimulated by the periostin peptide 142~151.

## Discussion

We have previously reported that periostin stimulates migration, adhesion, and tube formation of ECFCs through the first FAS-I domain-dependent mechanism [18]. In the current study, we characterized pro-angiogenic peptide sequences of the first FAS-I domain by serial deletion mapping and finally identified the periostin peptide 142~151 as a new pro-angiogenic peptide.

Since peptides can be utilized for regulating the physiological and biomedical functions of life, we focused on the identification of a pro-angiogenic peptide sequence from periostin. To discover the pro-angiogenic sequence, we have generated seven fragments of the first FAS-I domain of periostin. By serial deletion mapping of the periostin fragment, we could narrow down the first FAS-I domain to a minimal pro-angiogenic peptide sequence consisting of 20 amino acids. We have recently reported the development of neutralizing monoclonal antibodies against human periostin and identified that the sequence APSNEAWDNLDSDIRR within the first FAS-I domain of periostin, located at position from 136^th^ to 151^th^, was important [21]. Of note, the identified peptide sequence was covered by the recognition sequence of the neutralizing antibody, which inhibited the stimulation of migration and adhesion of ECFCs by the full length or the first FAS-I domain of periostin. These results support the importance of the periostin peptide 142~151 in the angiogenic potentials of periostin.

The periostin peptide 142~151 stimulated migration, adhesion, tube formation, and proliferation of ECFCs. *In vitro* data implicate a number of integrins in the regulation of cell growth, survival, and migration during angiogenesis. Periostin promotes the adhesion and migration of ovarian epithelial cells through the activation of α_v_β_3_ and α_v_β_5_ integrins and mediates the migration of vascular smooth muscle cells through the α_v_β_3_ and α_v_β_5_ integrins [12, 22]. Periostin-specific DNA aptamer disrupts interaction between periostin and its cell surface receptors, α_v_β_3_ or α_v_β_5_ integrins [23]. We have previously reported that integrin β3 and β5 subunits mediate the cell migration stimulated by the full length or the first FAS-I domain of periostin [18]. However, in the present study, we demonstrated a key role of integrin β5 subunit, but not β3 subunit, in the periostin peptide 142~151-mediated ECFC migration. These results raise a possible implication of another region of the first FAS-I domain in the periostin-induced activation of integrin β3. Because the periostin peptide 142~151 induced migration, adhesion, tube formation, and phosphorylation of Akt and Erk as potent as the full length of periostin, it is likely that the periostin peptide 142~151 plays a pivotal role in the regulation of angiogenic properties of ECFCs. These results may contribute to the development of novel therapeutics for tissue regeneration by stimulating ECFC-mediated angiogenesis.

## Supporting information

S1 Fig Identification of a novel peptide mapping in the first FAS 1 domain of periostin. (TIF)
