# Supplementary figures and images for: Identification of a novel angiogenic peptide from periostin
Source: PLoS One. 2017 Nov 2;12(11):e0187464. doi: 10.1371/journal.pone.0187464 (PMC5667812; doi:10.1371/journal.pone.0187464)

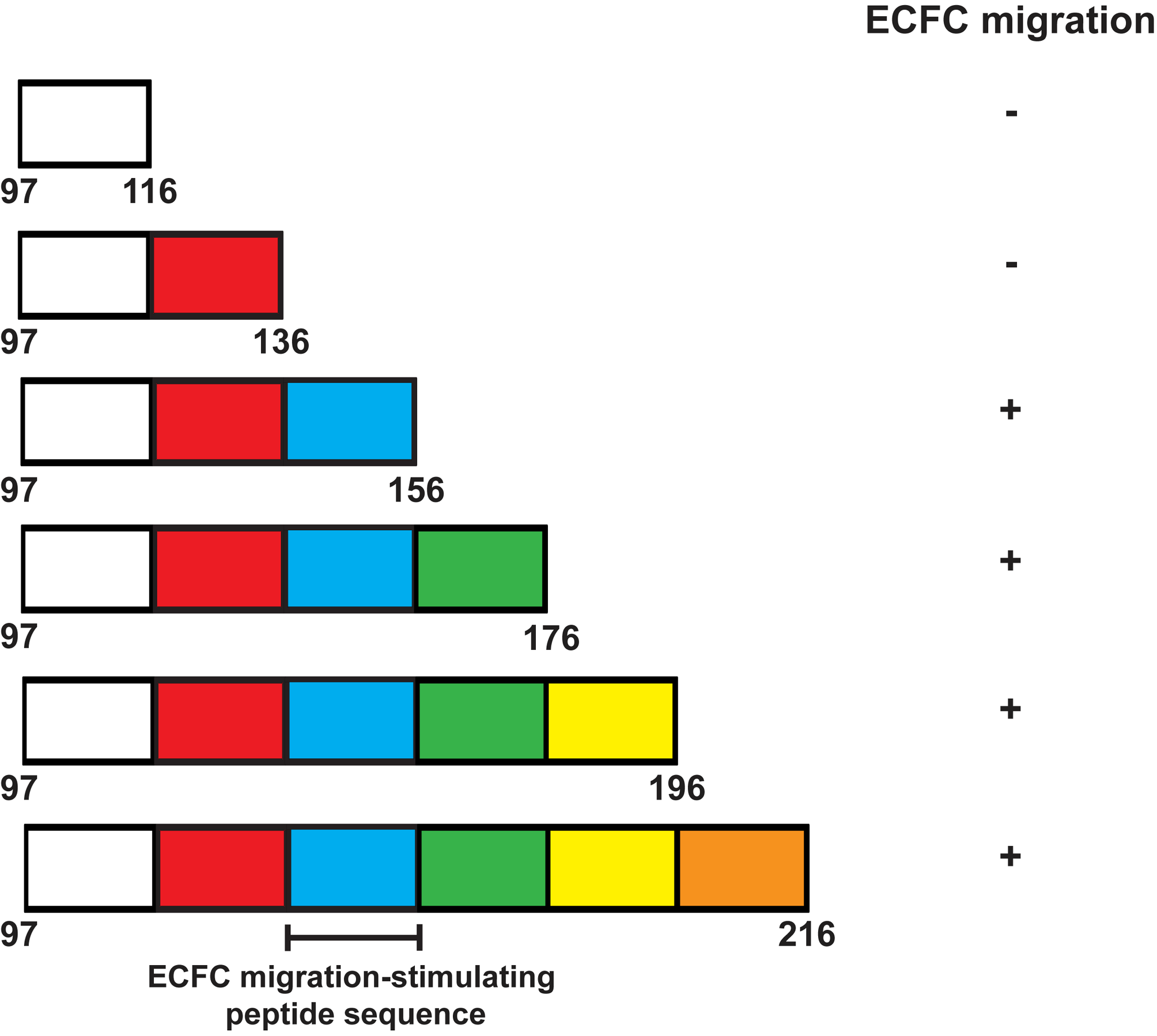

Supplement: S1 Fig — (TIF) [file pone.0187464.s001.tif]
